# 40.1 INFLAMMATORY CYTOKINES ARE ELEVATED IN THE MIDBRAIN IN SCHIZOPHRENIA

**DOI:** 10.1093/schbul/sby014.164

**Published:** 2018-04-01

**Authors:** Tertia Purves-Tyson, Debora Rothmond, Cynthia Shannon Weickert

**Affiliations:** Neuroscience Research Australia

## Abstract

**Background:**

Neuroinflammation is attractive as a candidate mechanism contributing to schizophrenia neuropathology. In ~40% of people with schizophrenia, pro-inflammatory cytokines are elevated in post mortem prefrontal cortex and in peripheral blood of living patients. Dopamine dysregulation contributes to cognitive deficits and psychosis and cytokines can increase dopamine production, yet post mortem midbrain cytokine transcripts have not been examined. We hypothesised that gene expression of inflammatory markers will be elevated in the midbrain of a subset of people that suffered with schizophrenia during their lives.

**Methods:**

Pro-inflammatory cytokine mRNAs for interleukin (IL) 1β, IL6, IL6 signal transducer (IL6ST), IL8, 1L18, tumor necrosis factor (TNF) α, SERPINA3, and the microglia marker, allograft inflammatory factor 1 (AIF1), were examined by qPCR in the midbrain from 28 schizophrenia cases and 29 healthy controls. All patients were on antipsychotics at time of death and antipsychotic medication was converted to chlorpromazine (CPZ) equivalents. Inflammatory subgroups were defined using two-step cluster analysis of cytokine mRNAs on the entire cohort. Chi-squared was used to test if the number of individuals in the inflammatory groups differed on the basis of diagnosis. Student’s t-tests or ANCOVA were used to detect diagnostic differences and differences between inflammatory/diagnosis subgroups. Student’s t-tests were used to compare CPZ equivalent doses in the low and high inflammation schizophrenia groups.

**Results:**

SERPINA3, IL1β, IL6 mRNAs were increased by more than 150% and IL6ST mRNA by 17% in the midbrain from schizophrenia patients compared to controls (F>4.0, p<0.0001–0.05), whilst IL8, IL18 and AIF1 mRNAs were unchanged (p>0.05). Cluster analysis revealed 13 individuals as high inflammation and 44 as low inflammation. All 13 individuals in the high inflammatory group were schizophrenia cases and the remaining 15 schizophrenia cases and all the control cases were low inflammation (χ2=57.0, P<0.0001, N=57). SERPINA3, IL6, IL1β and TNFα mRNAs were all increased in the high inflammation/schizophrenia compared to control and low inflammation/schizophrenia groups (p<0.002–0.05). AIF mRNA was not changed by diagnosis, but was increased in the high inflammation/schizophrenia compared to the low inflammation/schizophrenia group (p=0.015). The schizophrenia/high inflammation group received higher lifetime, daily and last CPZ equivalent doses (t(20–26)<-2.7, p<0.05) compared to the schizophrenia/low inflammation group.

**Discussion:**

Inflammatory markers were elevated in the midbrain in ~50% of schizophrenia cases, whilst no controls were classified as high inflammation. This data suggests that increases in pro-inflammatory cytokines extend to midbrain regions and may contribute to the neuropathology of the disorder by contributing to dopamine dysregulation. PET studies relate increased microglia activity to at-risk symptom severity in medication naïve people at ultra high risk for schizophrenia, we suggest that the higher dose of antipsychotics in the high inflammation group along with increased microglial marker may indicate that these patients were sicker and thus, required more medication, rather than antipsychotics increasing inflammatory markers. In conclusion, increased cytokine transcripts indicate a neuroinflammatory process in the midbrain in some people with schizophrenia. Future post mortem studies will explore whether previously identified changes in dopamine-related transcripts in the midbrain in schizophrenia are altered according to inflammatory state.

